# T Cells in Fibrosis and Fibrotic Diseases

**DOI:** 10.3389/fimmu.2020.01142

**Published:** 2020-06-26

**Authors:** Mengjuan Zhang, Song Zhang

**Affiliations:** College of Life Sciences, Nankai University, Tianjin, China

**Keywords:** T cells, fibrosis, fibrotic diseases, T helper, CD8, Treg, NKT, γδ T

## Abstract

Fibrosis is the extensive deposition of fibrous connective tissue, and it is characterized by the accumulation of collagen and other extracellular matrix (ECM) components. Fibrosis is essential for wound healing and tissue repair in response to a variety of triggers, which include infection, inflammation, autoimmune disorder, degenerative disease, tumor, and injury. Fibrotic remodeling in various diseases, such as liver cirrhosis, pulmonary fibrosis, renal interstitial fibrosis, myocardial infarction, systemic sclerosis (SSc), and graft-versus-host disease (GVHD), can impair organ function, causing high morbidity and mortality. Both innate and adaptive immunity are involved in fibrogenesis. Although the roles of macrophages in fibrogenesis have been studied for many years, the underlying mechanisms concerning the manner in which T cells regulate fibrosis are not completely understood. The T cell receptor (TCR) engages the antigen and shapes the repertoire of antigen-specific T cells. Based on the divergent expression of surface molecules and cell functions, T cells are subdivided into natural killer T (NKT) cells, γδ T cells, CD8^+^ cytotoxic T lymphocytes (CTL), regulatory T (Treg) cells, T follicular regulatory (Tfr) cells, and T helper cells, including Th1, Th2, Th9, Th17, Th22, and T follicular helper (Tfh) cells. In this review, we summarize the pro-fibrotic or anti-fibrotic roles and distinct mechanisms of different T cell subsets. On reviewing the literature, we conclude that the T cell regulations are commonly disease-specific and tissue-specific. Finally, we provide perspectives on microbiota, viral infection, and metabolism, and discuss the current advancements of technologies for identifying novel targets and developing immunotherapies for intervention in fibrosis and fibrotic diseases.

## Introduction

As a leading cause of mortality, fibrotic diseases can occur in virtually every organ and tissue. Extracellular matrix (ECM) component deposition is observed in many fibroproliferative diseases, including liver cirrhosis (LC), pulmonary fibrosis, renal interstitial fibrosis, myocardial infarction, systemic sclerosis (SSc), and graft-versus-host disease (GVHD) (1, 2). Numerous studies have demonstrated that the immune response plays an essential role in fibrosis and fibrotic diseases. Systematic investigation of the immune cells and signaling pathways remains fundamental for developing novel therapies (3). Therefore, it is essential to understand the key factors influencing fibrosis.

After hepatic injuries, immune cells participate in wound healing and tissue repair by initiating inflammation. The infiltrated T cells, macrophages, neutrophils, dendritic cells (DCs), and the liver-resident macrophages, Kupffer cells, cooperatively contribute to the liver fibrotic cascade and lead to the activation of hepatic stellate cells (HSCs) and the generation of myofibroblasts (4, 5). Non-alcoholic fatty liver disease (NAFLD) is characterized by hepatic steatosis with the presence of T cells, including natural killer T (NKT) cells, γδ T cells, CD8^+^ cytotoxic T lymphocytes (CTL), regulatory T (Treg) cells, and T helper cells. These T cells exert their function by attenuating or aggravating the liver injury and fibrosis progression (6).

Further, pulmonary fibrosis is a highly lethal pathological process, in which T cell responses contribute to the pathogenesis of idiopathic pulmonary fibrosis (IPF), cystic fibrosis (CF), and various other lung diseases (7, 8). However, the functions of each T cell subset appear to be perplexing and are influenced by interactions with epithelial cells or fibroblasts, the pulmonary localization of fibrosis in the bronchial or alveolar region, and the disease progression stage (9, 10). T cells can regulate the pulmonary fibrosis outcome through cAMP-regulated chloride channels (11), Fas-Fas ligand (FasL) interactions (12), or T cell exhaustion (13). Given the strong relationship between fibrosis and inflammation, the determination of precise functions of T cells may also be beneficial for inflammatory and autoimmune diseases.

Cardiovascular disease (CVD) is a class of heart or blood vessel-related disease. T cells modulate cardiac fibroblasts and MMP activity during cardiac fibrosis and hypertension (14–16). Using T cell-deficient mice disease model, previous studies have demonstrated the pivotal role of T cells in heart failure (HF) (17, 18), myocardial fibrosis (19), ischemia (20), and myocardial infarction (21).

Furthermore, intestinal fibrosis occurs in the gastrointestinal tract during chronic diseases such as Crohn's disease (CD), ulcerative colitis (UC), ulcerative jejunoileitis, and radiation enteritis. T cells participate in persistent dysregulated inflammation and lead to excessive myofibroblast proliferation, ECM deposition, and scar tissue formation (15, 22).

Systemic sclerosis (SSc) affects the skin and multiple internal organs, causing excessive ECM deposition and vasculopathy. Many studies have suggested that some T cell subsets, such as Th17, Treg, Th2, Th9, and Th22, can serve as a hallmark of SSc. Therefore, discussing the mechanisms of each T cell subset may help identify leash the pro-fibrotic T cells and cytokines in SSc (2, 23).

Renal fibrosis is considered to be a consequence of immune response involving myofibroblast accumulation and matrix deposition. Researchers have observed that T cell activation and infiltration can cause interstitial fibrosis and glomerular injury in the kidney (24).

Furthermore, GVHD patients may suffer vascular injury caused by the immune response between recipient endothelial cells and circulating alloreactive donor T cells. T helper cells, including Th17 and Tfh cells, secrete IL-17 and IL-21 cytokines and augment this immune response and fibrosis outcome (25).

T cell-dependent fibrosis plays a crucial role in muscle regeneration, and sclerotic scar tissue in muscles is attributed to Duchenne muscular dystrophy (DMD). Although T cells can undermine the muscle regeneration (26), they are also important for biomaterial scaffold muscle tissue repair (27).

**Figure 1:**
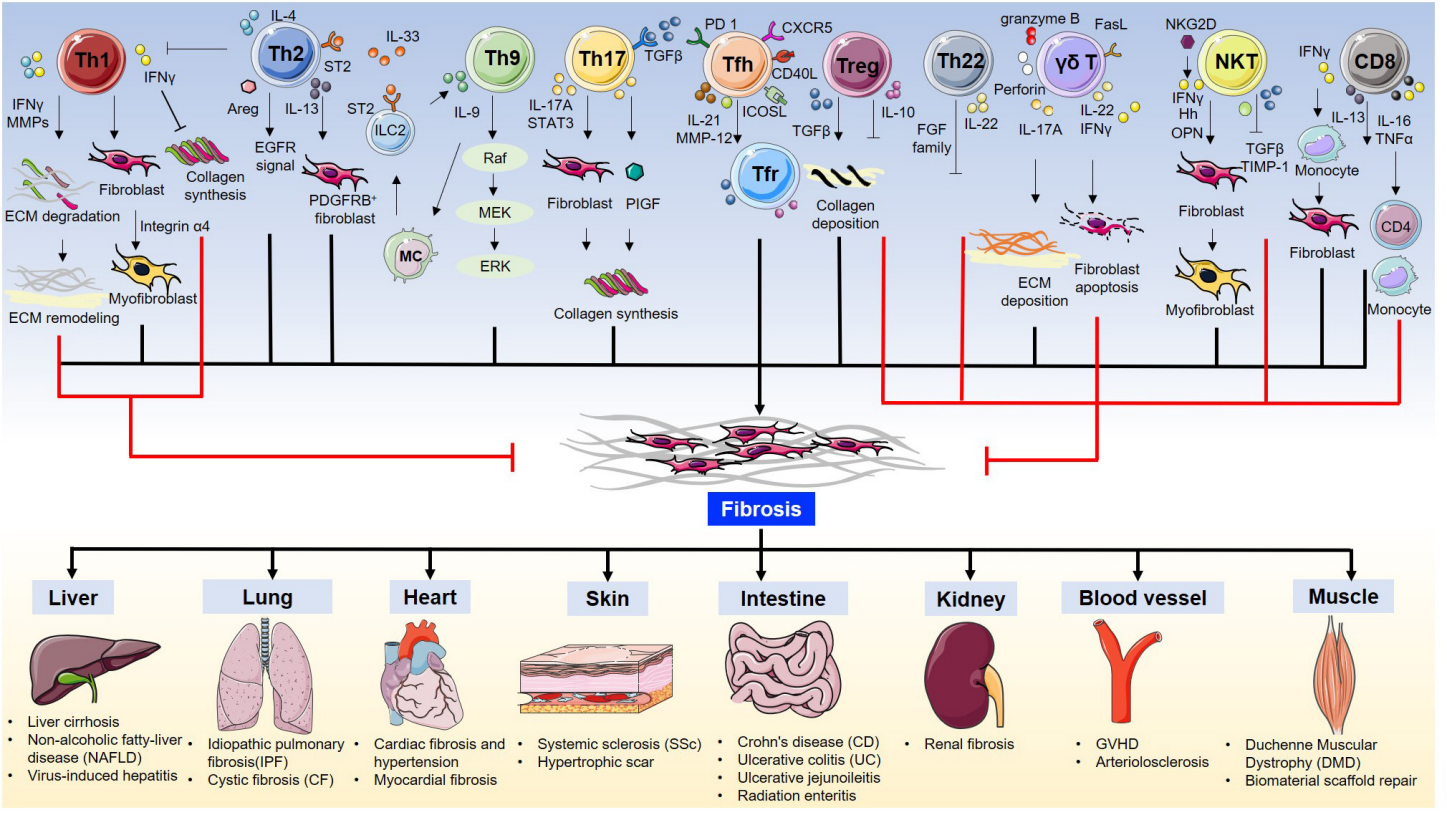
T cells in fibrosis and fibrotic diseases.

In summary, T cells are essential for fibrosis and fibrotic diseases. The orchestration of fibrotic tissue remodeling is programed by multiple cytokines, chemokines, and growth factors (28, 29). In this review, we briefly discuss the role of all T cell subsets and their underlying mechanisms in fibrosis and fibrotic diseases (Table 1, Figure 1).

**Table 1:** The role of T cells in fibrosis and fibrotic diseases.

**T cell subset**	**Tissue/organ**	**Species**	**Disease**	**Effect in fibrosis**	**References**
Th1	Peritoneal membrane	Mouse	SES-induced inflammation	Pro-fibrotic	(30)
Th1	Heart	Human Mouse	Cardiac fibrosis	Pro-fibrotic	(31)
Th2	Lung	Human	Cystic fibrosis	Pro-fibrotic	(32)
Th2	Lung	Human	Cystic fibrosis	Pro-fibrotic	(33)
Th2	Lung	Human Mouse	ECRS HDM	Pro-fibrotic	(34)
Th17	Liver	Mouse	BDL,CCl_4_	Pro-fibrotic	(35)
Th17	Lung	Mouse	*S. rectivirgula*-induced lung fibrosis	Pro-fibrotic	(36)
Treg	Lung	Mouse	Bleomycin-induced pulmonary fibrosis	Anti-fibrotic	(37)
Treg	Heart	Mouse	Angiotensin II-infused hypertension	Anti-fibrotic	(38)
Treg	Lung	Mouse	Silica-induced lung fibrosis	Pro-fibrotic	(39)
Treg	Heart	Mouse	MI	Pro-fibrotic	(40)
Treg	Lymphatic tissues	Macaque	SIV infection	Pro-fibrotic	(41)
Treg	Liver	Human	Chronic HCV	Anti-fibrotic	(42)
Treg	Liver	Humanized mouse	HIV-1 infection	Anti-fibrotic	(43)
Treg	Liver	Mouse	Biliary fibrosis in murine sclerosing cholangitis	Anti-fibrotic	(44)
Treg	Lung	Mouse	*A. fumigatus*-induced lung fibrosiss	Anti-fibrotic	(45)
Treg	Kidney	Mouse	IRI	Anti-fibrotic	(46)
Tfh	Liver	Mouse	*S. japonicu*m infection	Pro-fibrotic	(47)
Tfh	Skin	Human Mouse	SSc GvHD	Pro-fibrotic	(48)
Tfh	Lung	Human	PBC	Pro-fibrotic	(49)
Tfh	Lung	Human	IPF	Pro-fibrotic	(50)
Th9	Lung	Mouse Human	Silica-induced lung fibrosis IPF	Pro-fibrotic	(51)
Th9	Liver	Mouse	CCl_4_	Pro-fibrotic	(52)
Th9	Lung	Human	Cystic fibrosis	Pro-fibrotic	(53)
Th22	Heart	Mouse	CVB3 infection	Anti-fibrotic	(54)
Th22	Lung	Human	Cystic fibrosis	Anti-fibrotic	(55)
Th22	Liver	Mouse	CCl_4_	Anti-fibrotic	(56)
Th22	Liver	Mouse	MCD diet	Anti-fibrotic	(57)
CD8	Microvessels in the skin	Human	GVHD	Pro-fibrotic	(58)
CD8	Thyroids	Mouse	Thyroid epithelial cell fibrosis	Pro-fibrotic	(59)
CD8	Lung	Mouse	Bleomycin-induced pulmonary fibrosis	Pro-fibrotic	(60)
CD8	Kidney	Mouse	UUO	Anti-fibrotic	(61)
NKT	Lung	Mouse	Bleomycin-induced pulmonary fibrosis	Anti-fibrotic	(62)
NKT	Liver	Mouse	TAA, CCl_4_	Pro-fibrotic	(63)
NKT	Liver	Mouse	α-GalCer, CCl_4_	Pro-fibrotic	(64)
NKT	Liver	Mouse Human	NASH	Pro-fibrotic	(65)
NKT	Liver	Mouse	CCl_4_	Pro-fibrotic	(66)
NKT	Liver	Mouse	PBC	Pro-fibrotic	(67)
NKT	Liver	Mouse	NASH	Pro-fibrotic	(68)
NKT	Liver	Mouse	CCl_4_	Anti-fibrotic	(69)
NKT	Liver	Mouse	CCl_4_, MCD diet	Pro-fibrotic	(70)
NKT	Lung	Human	ILDs	Anti-fibrotic	(71)
γδ T	Lung	Human	IPF	Anti-fibrotic	(72)
γδ T	Lung	Human	Cystic fibrosis	Anti-fibrotic	(73)
γδ T	Liver	Mouse	CCl_4_, MCD diet	Anti-fibrotic	(74)
γδ T	Lung	Mouse	Bleomycin-induced pulmonary fibrosis	Anti-fibrotic	(75)
γδ T	Lung	Human	Cystic fibrosis	Anti-fibrotic	(76)
γδ T	Lung	Mouse	Bleomycin-induced pulmonary fibrosis	Anti-fibrotic	(77)
γδ T	Lung	Mouse	*B. subtilis*-induced pulmonary fibrosis	Anti-fibrotic	(78)
γδ T	Lung	Mouse	Bleomycin-induced pulmonary fibrosis	Anti-fibrotic	(79)
γδ T	Kidney	Mouse	UUO	Pro-fibrotic	(80)
γδ T	Liver	Mouse	CCl_4_	Pro-fibrotic	(81)
γδ T	Liver	Mouse	CCl_4_	Anti-fibrotic	(82)
γδ T	Liver	Mouse	*S. japonicum* infection	Pro-fibrotic	(83)
γδ T	Kidney	Human	Tubulointerstitial fibrosis	Pro-fibrotic	(84)

*SES, Staphylococcus epidermidis; ECRS, eosinophilic chronic rhinosinusitis; HDM, house dust mite; BDL, bile duct ligation; CCl_4_, carbon tetrachloride; IPF, idiopathic pulmonary fibrosis; MI, myocardial infarction; SIV, simian immunodeficiency virus; IRI, ischemia/reperfusion injury; SSc, Systemic sclerosis; GVHD, graft-versus-host disease; PBC, primary biliary cirrhosis; CVB3, Coxsackie virus B3; MCD, methionine-choline–deficient; UUO, unilateral ureteric obstruction; TAA, thioacetamide; NASH, nonalcoholic steatohepatitis; ILDs, interstitial lung diseases*.

## The Role and Mechanisms of T Cells in Fibrosis and Fibrotic Diseases

### Th1 Cells and Fibrosis

Inflammation is considered to be one of the major steps leading to fibrosis (29). However, the production of pro-inflammatory cytokines is not always pro-fibrotic. IL-12 induces the differentiation of naïve CD4 cells to Th1 cells to produce the pro-inflammatory cytokine IFNγ. IFNγ suppresses fibroblast-induced collagen synthesis and attenuates fibrosis. Therefore, Th1 cells are largely considered to play an anti-fibrotic role (85). Wynn et al. previously used IL-12 for treating *Schistosoma mansoni* infection in mice. This not only inhibited Th2-dominated immune response by elevating Th1 cytokine expression but also drastically ameliorated fibrosis (86). IFNγ production up-regulates the expression of matrix metalloproteinases (MMPs), including MMP-2, MMP-7, MMP-9, and MMP-13, to degrade ECM components. This proteolytic activity helps alter ECM remodeling and ameliorates fibrosis (87). Th1 cells and cytokine IFNγ are not always anti-fibrotic. On the contrary, they can also play a harmful role in bone regeneration (88), liver injury (89), and fibrotic diseases (30, 31). In cardiac fibrosis, Th1 cell infiltration leads to the activation of cardiac fibroblasts (CFBs) which then transform into myofibroblasts via integrin alpha4. Further, Th1 cells induce TGFβ expression in myofibroblasts, which forms a fibrillary ECM in the myocardium (31).

### Th2 Cells and Fibrosis

Th2 cells are characterized by the production of signature cytokines IL-4, IL-5, and IL-13. Th2 cells, along with eosinophils, basophils, macrophages, and type 2 innate lymphoid cells (ILC2), contribute to the type 2 immunity-induced pathological process of fibrosis (90). As a commonly recognized opponent of Th1 cells, Th2 cells can alter Th1-associated IFNγ expression levels. In *Pseudomonas aeruginosa*-infected cystic fibrosis patients, an elevated ratio of pulmonary CCR4^+^ Th2 cells to CXCR3^+^ Th1 lymphocytes was found in the bronchoalveolar lavage fluid, with significantly higher levels of Th2 cytokines IL-4 and IL-13 (32). In addition to infection, allergic inflammation also triggers a Th2 response. Asthma is a chronic allergic inflammatory disease with fibrotic airway remodeling. However, the mechanism underlying airway fibrosis remains poorly understood. Morimoto et al. found that IL-33 induces the production of ST2^hi^ memory-type pathogenic Th2 cells which enhance amphiregulin (Areg) levels. Furthermore, amphiregulin-epidermal growth factor receptor (EGFR) signal results in osteopontin-producing eosinophils and fibrotic responses. Their study highlighted how Th2 memory cells are critical for allergy-induced airway fibrosis (34). It has been reported that aberrant and spontaneous development of Th2 cells in the lamina propria of TRAF6-knockout mice with eosinophilic enteritis causes fibrosis in the small intestine (91). Further, IL-13 is considered to be an essential Th2 cytokine for fibrosis (90). By specifically disrupting IL-13 signaling in liver-resident tissue fibroblasts, also known as hepatic stellate cells (HSCs), Gieseck et al. demonstrated that PDGFRB^+^ fibroblasts are necessary for mediating IL-13-induced pathological fibrosis in mice (92). Furthermore, IL-13-regulated lipogenesis, bile acid synthesis, and biliary-dependent steatosis seem to be distinct cellular pathways from fibrosis, suggesting the possible intervention of IL-13 for the promotion of hepatobiliary expansion without aggravating fibrosis (92). Th2-targeted treatment has been tested in fibrosis-related diseases. For example, vitamin D3 was found to attenuate Th2 response in cystic fibrosis patients with allergic bronchopulmonary aspergillosis by substantially reducing DC-expressed costimulatory molecule OX40 ligand (OX40L) and increasing TGFβ expression (33).

### Th9 Cells and Fibrosis

Th9 cells were originally described in parasitic infections and allergic diseases. As a newly defined subset of T helper cells, it responds to environmental cues and cytokine milieu to produce IL-9 (93). Pleiotropic cytokine IL-9 activates various target cells including dendritic cells, mast cells, and CD8^+^ T cells, and is involved in the pathological processes of multiple diseases including inflammatory diseases, infectious diseases, autoimmune diseases, and cancer (93–95). Elevated serum IL-9 levels have been reported in patients with periportal fibrosis caused by *Schistosoma mansoni* infection (96). In both the silica-induced lung fibrosis mouse model and human patients with idiopathic pulmonary fibrosis (IPF) and cystic fibrosis, IL-9 levels were found to be correspondingly elevated (51, 53). While the administration of IL-9 neutralizing antibody protects mice from IPF and cystic fibrosis (CF) (51, 53). During an infection event, IL-33 activates ILC2 to produce IL-9, and the Th9 cells and IL-9 cytokine production is further amplified by IL-9-activated mast cell-driven ILC2 expansion (53). In liver cirrhosis patients, IL-9 is also significantly increased and has been proven to play an important role in hepatic fibrosis progression. IL-9 has further been reported to activate Raf/MEK/ERK signaling pathway in a commonly used carbon tetrachloride (CCl_4_)-induced liver fibrosis mouse model. Consistent with studies in lung fibrotic diseases, IL-9 antibody inactivates hepatic stellate cells (HSCs) and ameliorates liver fibrosis (52). These results offer a possible treatment strategy for reducing fibrosis by blocking IL-9 signaling.

### Th17 Cells and Fibrosis

Th17 cells have been discovered and characterized as the third subset of T helper cells. Characterized by their IL-17-producing ability, Th17 cells are critically involved in inflammatory responses (97). The relation between Th17 cells and fibrosis has been investigated in recent years. Th17 cells serve as a key component in mucosal immunity including the respiratory tract (98). In a hypersensitivity pneumonitis mouse model established by repeated exposure to *Saccharopolyspora rectivirgula*, increased Th17 were responsible for the inflammatory and fibrotic responses (36). Similarly, Th17 cells infiltrated into the bronchial submucosa of human patients with cystic fibrosis (CF) (99, 100). In the liver, IL-17 targets multiple types of cells, including Kupffer cells and hepatic stellate cells (HSCs). Gao et al. observed increased levels of IL-17A and IL-17 receptor in a liver fibrosis model induced by intragastric gavage with CCl_4_ or bile duct ligation (35). Mechanistically, IL-17 activates the STAT3 signaling pathway in HSCs to produce type 1 collagen. Interestingly, the deletion of IL-23, or administration of IL-17E (also known as IL-25), attenuates liver fibrosis (35). Given the fact that IL-17 is not only produced by Th17 but also by γδ T cells (101), NKT cells (102), and type 3 innate lymphoid cells (ILC3) (103), and the differentiation and function of Th17 cells highly rely on the cytokine milieu, such as IL-1β, IL-6, IL-23, and TGFβ (104), it is important to identify novel factors that may regulate Th17 cell differentiation in fibrosis-related diseases. Angiogenesis contributes to fibroproliferative diseases. Recently, Jameson et al. reported that placental growth factor (PlGF), an angiogenic factor of the VEGF family, is specifically secreted from Th17 cells. PIGF is also required for the progression of collagen-induced arthritis in mice (105). Th17 cell differentiation program is highly heterogeneous (106) and is controlled by numerous factors, including febrile temperature (107). The fever shapes Th17 cell differentiation by SUMOylation of the transcription factor SMAD4 (107), which is tightly associated with T cell function during inflammation (108, 109). SMAD4 has been reported to control Th17 function with an oncoprotein SKI (110, 111), which has been linked to wound healing and fibrosis (112). This evidence suggests that TGFβ signaling in T cells also plays an important role in regulating inflammatory responses (104). TGFβ controls fibrosis in different cell types. Elevated mechanical tension activates the TGFβ signaling loop in mouse alveolar stem cells (AT2) and causes progressive pulmonary fibrosis (113). The complexity of the cytokine regimen responsible for Th17 differentiation may be harnessed to treat fibrotic diseases.

### Th22 Cells and Fibrosis

Th22 cells are characterized by the production of cytokine IL-22. Although there exists a Th17-expressed IL-22 population, the Th22 cells exhibit transcriptomes distinct from those of Th17 cells (114). Importantly, Th22 cells express several members of the fibroblast growth factor (FGF) family, such as FGF1, FGF-5, FGF-12, and FGF-13, which are master regulators for wound healing, tissue repair, tissue regeneration, and fibrosis (114). Th22 represents a key T cell subset for epidermal immunity, and its levels are significantly increased in the epidermis of patients with morphea and SSc (115). Th22 cells are also increased in patients with cystic fibrosis in response to *P. aeruginosa* infection; this suggests the involvement of Th22 cells in pulmonary immunity (55). Th22 cells are also highly associated with liver fibrosis and are considered to play a hepatoprotective role (116). Th22 cell levels are elevated in mice with CCl_4_-induced liver fibrosis (56), mice with methionine choline-deficient (MCD) diet-induced non-alcoholic steatohepatitis (NASH)(57), and human patients with liver cirrhosis (LC) (117). Liver-infiltrated Th22 cells, or recombinant IL-22 treatment, ameliorates fibrogenesis by attenuating hepatic stellate cell activation (56). Similarly, increased levels of Th22 and IL-22 are observed in mice with Coxsackie virus B3 (CVB3)-induced chronic myocarditis and dilated cardiomyopathy. Consistently, the expression of matrix metalloproteinase-9 (MMP9) is increased and that of metalloproteinase-1 (TIMP-1) inhibitor is decreased (54). Furthermore, the administration of an IL-22-neutralizing antibody exacerbated myocardial fibrosis as well as mortality. It is important to note that although IL-22 is mainly produced by immune cells including Th22 cells, Th17 cells, γδ T cells, NKT cells, and type 3 innate lymphoid cells (ILC3), it primarily targets non-immune cells (118). These facts suggest that targeting crosstalk between immune cells and tissues via the IL-22 signaling pathway is possible in fibrogenesis.

### Regulatory T Cells and Fibrosis

Regulatory T cells (Tregs) play a pivotal role in modulating self-tolerance and immune homeostasis. It has been reported that cystic fibrosis patients with *P. aeruginosa* infection have impaired Tregs (119). Further, depletion of Treg cells by anti-CD25 antibody in silica-induced lung fibrosis attenuates fibrosis, and this process is probably dependent on the indirect function of Treg-secreted IL-10 and TGFβ (39). However, intranasal administration of TGFβ1-expressing plasmid results in increased TGFβ1- and IL-10-producing Treg cells and ameliorates bleomycin-induced lung fibrosis in mice (37). Recently, Ichikawa et al. found that depletion of CD69^hi^CD103^hi^Foxp3^+^ Treg cells resulted in substantially higher levels of lung fibrosis in mice exposed to *Aspergillus fumigatus* because of the pathology promoted by tissue-resident CD103^lo^CD44^hi^CD69^hi^CD4^+^ T cells, which express high level of fibrosis-related genes. Their work thus defined a new tissue-resident Treg subpopulation in the lungs (45). The role of Tregs in fibrosis can be controversial and may be associated with a specific type of disease model. In angiotensin II-infused hypertensive mice, the adoptive transfer of CD4^+^CD25^+^ Treg cells improves cardiac hypertrophy and ameliorates cardiac fibrosis (38). However, in mice with ischemic cardiomyopathy, Treg ablation alleviates hypertrophy and cardiac fibrosis (40). Under the context of viral infections, TGFβ1^+^ Treg cells induce deleterious collagen deposition and lymphatic tissue fibrosis in simian immunodeficiency virus (SIV)-infected rhesus macaques, showing a pro-fibrotic effect (41). However, in hepatitis C virus (HCV)-infected human patients, or in human immunodeficiency virus type 1 (HIV-1)-infected humanized mice, Treg cells prevent liver immunopathogenesis and limit liver fibrosis (42, 43). Treg cells exhibit different transcriptional changes in response to regenerative or fibrogenic environmental cues. During kidney fibrosis, remarkably increased levels of tissue-resident Treg cells express elevated fibrosis-related transcription factors, such as *Id2, Nf*κ*b, Rgs2*, and *Junb* (46). Depletion or restoration of Treg cells may become a viable approach in controlling fibrosis in this case. In contrast, in Mdr2 (Abcb4) deficient mice with sclerosing cholangitis, a low dose of IL-2 treatment diminishes biliary injury and fibrosis by the expansion of intrahepatic Tregs (44).

### Tfh Cells and Fibrosis

T-follicular helper (Tfh) cells, characterized by the expression of the lineage-specific transcription factor Bcl6 and production of IL-21, are essential for B cell function. They express high level of surface markers, such as CXCR5, CD40L, ICOS, and PD-1 (120). Upon *Schistosoma japonicum* infection, macrophages drive the differentiation of Tfh cells through CD40-CD40L and ICOS-ICOSL interactions. Following this, the infiltrated Tfh cells increase the hepatic granuloma formation and lead to severe liver fibrosis (47). The levels of these cell are also increased in patients with primary biliary cirrhosis (PBC) (49). In patients with idiopathic pulmonary fibrosis, the levels of CXCR5^+^ICOS^+^PD-1^+^ Tfh cells are increased in the peripheral blood (50). Further, the levels of CXCR5^+^ICOS^+^PD-1^+^ Tfh cells are strongly associated with dermal fibrosis in patients with systemic sclerosis (SSc). A mouse sclerodermatous GVHD (GVHD-SSc) model suggested that the level of these profibrotic Tfh cells are IL-21- and MMP-12-dependent. Furthermore, it has been shown that both IL-21 and ICOS antibody administration can effectively reduce skin fibrosis (48). As a new subset of T cells, Tfh cells may bring novel insights into fibrotic disease therapies.

### Tfr Cells and Fibrosis

T follicular regulatory (Tfr) cells, sharing Tfh makers including CXCR5, Bcl6, ICOS, and PD-1, are characterized by Foxp3 expression and have a regulatory function (121). In patients with primary biliary cholangitis (PBC), the levels of CD4^+^CXCR5^+^CD127^lo^CD25^hi^ Tfr cells, as well as CCR7^hi^PD-1^lo^ central memory Tfr cells, are dramatically decreased, whereas those of CCR7^lo^PD-1^hi^ effector memory Tfr cells are increased. This evidence suggests the involvement of Tfr cells in primary biliary cholangitis regulation (122). In patients with chronic hepatitis B (CHB) infection, levels of circulating Tfr cells are significantly increased and are positively associated with FIB-4, which is the fibrosis index based on four factors (123). Another study including patients with CHB and chronic hepatitis C (CHC) also indicated that Tfr cells possibly modulated liver fibrosis by secreting the regulatory cytokine IL-10 and TGFβ (124). Thus, evidence suggests an emerging role of Tfr cells in virus-induced liver fibrosis (123).

### Cytotoxic T Cells (CTLs, CD8^+^ T Cells) and Fibrosis

Cytotoxic T cells (CTLs, CD8^+^ T cells), expressing CD8 glycoprotein as an identity marker, are vital for killing infected cells and tumor cells. They also play important roles in many fibrosis-related diseases. Perivascular infiltrated CD8^+^ T cells are found in patients with GVHD of the skin (58). In a mouse model of acute cerebral ischemia, CD8^+^ T cells infiltrated into the perivascular space and expressed IL-16 to recruit monocytes and CD4^+^ T cells, resulting in reduced hindlimb muscle fibrosis (125). Furthermore, in a renal fibrosis model, CD8^+^ T cells and IFNγ reduced the CD4^+^ T cell-induced monocyte-to-fibroblast transition (61). Activated CD8^+^ T cells can produce TNFα and induce thyroid fibrosis (59). They can also secrete IL-13 to mediate bleomycin-induced pulmonary fibrogenesis in an IL-21-dependent manner (60). In contrast, fibrosis also has an impact on the immunosurveillance functions of CD8^+^ T cells. For example, mouse liver fibrosis during HBV infection can jeopardize hepatocellular antigen recognition by intravascular CD8^+^ T cells when crawling along liver sinusoids (126). Further, liver fibrosis and non-alcoholic steatohepatitis (NASH) lead to the accumulation of liver resident IgA^+^PD-L1^+^IL-10^+^ cells that directly impair CTL/CD8^+^ T cell functions against tumor-associated antigens, resulting in the development of hepatocellular carcinoma (HCC) (127). The fibrosis of secondary lymph nodes may result in CD8^+^ T cell depletion after vaccine responses (128). Therefore, while developing vaccine strategies and triggering pro-fibrotic inflammatory responses for infectious diseases, the impact on CD8^+^ T cells must be considered.

### NKT Cells and Fibrosis

Natural killer T (NKT) cells express the αβ T cell receptor and recognize the glycolipid antigens presented by MHC I-liked protein CD1d. Being largely presented in the liver, NKT cells are central components of the immune response during liver injury, repair, inflammation, and fibrosis (129–131). A xenobiotics-induced liver fibrosis model is commonly used for determining the effect of NKT cells on liver fibrogenesis. NKT cells are critically involved in liver fibrosis induced by thioacetamide (TAA) (63), CCl_4_ (63, 64, 66, 69), α-galactosylceramide (α-GalCer) (64), or 3,5-diethoxycarbonyl-1,4-dihydrocollidine (DDC) (69). It seems that the function of NKT cells in fibrosis is subject to different contexts. In TAA-induced liver fibrosis, the CD1d deficient mice show ameliorated liver fibrogenesis with blunted TIMP-1 expression (63), whereas in CCl_4_-induced liver fibrosis, NKT-deficient mice are more susceptible (64). Furthermore, infiltrated NKT cells increase NKG2D ligand expression to activate HSCs and ameliorate liver fibrosis (66, 69). The participation of NKT cells at different fibrosis stages may also cause divergence (64). In a methionine choline-deficient (MCD) diet-induced non-alcoholic steatohepatitis (NASH) model, NKT cells accumulated, Hedgehog (Hh) and osteopontin (OPN) levels increased, and HSCs activated and differentiate to myofibroblasts (65, 68). Additionally, in a xenobiosis-induced model of primary biliary cirrhosis (PBC), the invariant natural killer T cell activator α-GalCer induced exacerbated fibrosis (67). Moreover, it has been reported that IL-30 treatment (69) or targeting CXCR6/CLCL16 (70) may serve as a potential approach for the amelioration of liver fibrosis. Further, NKT cells play a role in lung diseases (62, 71). In a bleomycin-induced pulmonary mouse fibrosis model, the genetic deletion of CD1d was reported to result in severe fibrosis, although adoptive transfer of NKT cells protected the mice from fibrosis. NKT cells attenuate pulmonary fibrosis by producing IFNγ and reducing TGFβ levels (62). Given the unique characteristics of NKT cells, the precise mechanism for NKT cells in fibrosis warrants further investigation.

### γδ T Cells and Fibrosis

γδ T cells are unconventional T cells characterized by a T cell receptor γ chain and δ chain, which are not restricted by MHC. These cells may either instigate fibrosis or resolve fibrosis in a cytokine-dependent and disease-dependent manner (132). Although the overall number of γδ T cells is small, these cells represent a major T cell population in the skin, gut, and lung. The dendritic γδ T cells in the dermis are also named DETC, dendritic epidermal T cells. Further, it has been reported that skin fibroblasts exhibit higher proliferative activity and stronger collagen synthesis ability when cultured with γδ T cell supernatant (133). Studies on patients with systemic sclerosis have demonstrated the involvement of γδ T cells (134–136). The level of pathogenic CD27^+^ γδ T cells were reported to increase, with upregulation of granzyme B or perforin expression (134). Further, Vγ9Vδ2, a subset of human γδ T cells, shows anti-fibrotic potential with the production of IFNγ (135, 136).

An abundance of approximately 8–20% γδ T cells in resident pulmonary lymphocytes shows the possible engagement of γδ T cells in lung infection, chronic inflammation, and subsequent pulmonary fibrosis (137). Sarcoidosis and idiopathic pulmonary fibrosis have shown to cause an increase in γδ T cell levels in the bronchoalveolar lavage fluid of patients (72). γδ T cells are also expanded in both cystic fibrosis patients with *P. aeruginosa* infection and mice with *Bacillus subtilis* infection (76, 138). In a bleomycin-induced fibrosis model, ablation of γδ T cells results in severe pulmonary fibrosis. Moreover, γδ T cells may prevent fibrosis by expressing CXCL10 (79). An important response of γδ T cells to infection and injury is the production of cytokine IL-17. Thus, γδ T cells were found to be the predominant source of IL-17 in mice with fibrosis induced by bleomycin (77), *B. subtilis* (139), and silica (140). IL-22 also contributes to the inhibition of pulmonary fibrosis. Deficiency in aryl hydrocarbon receptor (AhR) signaling or IL-22 enhances collagen deposition and accelerates fibrosis. Vγ6Vδ1 γδ T cells are the predominant source of IL-22 in protecting the lung from pulmonary fibrosis (78). Another major cytokine produced by γδ T cells is IFNγ. In a bleomycin-induced pulmonary fibrosis model, IFNγ produced by γδ T cells was found to attenuate fibrosis by indirectly inhibiting IL-17-secreting Th17 cells (75).

γδ T cells are also enriched in the liver and play an important role in liver fibrosis and cirrhosis (141). IL-17-producing γδ T cells are crucial in a variety of liver diseases (101). The chemokine receptor CCR6 is required for generating IL-17-producing γδ T cells. Hepatic γδ T cells contact and promote the FasL-induced HSC apoptosis to protect the liver from excessive fibrosis in an IL-17-dependent manner (74). IFNγ-producing γδ T cells are also protective in liver fibrosis by showing direct cytotoxicity against activated HSCs (82). Furthermore, hepatocyte-derived exosomes may mediate the activation of TLR3, which in turn enhances the production of IL-17 in γδ T cells (81). IL-17-producing γδ T cells are also enriched in patients with biliary atresia (BA), which is characterized by the destruction of the biliary system and liver fibrosis (142).

In addition, γδ T cells also display critical roles during fibrosis of other tissues. As γδ T cell is a key source of IL-17, the number of γδ T cells is elevated in patients with tubulointerstitial fibrosis, as determined by their biopsies (84). Further, in mice with kidney obstructive injury, γδ T cells are a major source of IL-17 and contribute to the pathogenesis of renal fibrosis with myofibroblast activation and ECM deposition (80). Moreover, in another study on a myocardial infarction mouse model, the IL-17-producing γδ T cells were found to promote fibroblast proliferation and aggravate fibrosis (143).

The aforementioned studies suggest that γδ T cells may be protective or deleterious in fibrosis in a cytokine-specific and tissue-specific manner. Thus, understanding the roles and underlying mechanisms of γδ T cells in the pathogenesis of fibrotic diseases would be useful for developing γδ T cell-based immunotherapies.

## Concluding Remarks and Perspectives

As a key process of wound healing, a variety of immune cells engage in the manifestation of inflammation and fibrosis. To date, the involvement of T cells has been well-established in orchestrating the fibrous tissue microenvironment. Although extensive studies have been conducted on T cells and fibrosis, many questions remain elusive.

What is the initial trigger of fibrosis? How do we identify risk factors leading to a severe fibrotic disease? How do we utilize T cells in other fibrosis-related narratives? The pandemic of 2019 coronavirus disease COVID-19, caused by the SARS-CoV-2 virus infection, has caused worldwide mortality (144). COVID-19 is accompanied by fibrosis (145, 146), and is particularly dangerous for patients with pulmonary fibrotic diseases including cystic fibrosis (147). Pulmonary fibrosis is also commonly developed in patients with other virus-induced respiratory diseases, including severe acute respiratory syndrome coronavirus (SARS-CoV) (148–150) and Middle East respiratory syndrome (MERS) (151). A better understanding of cellular and molecular mechanisms will help treat fibrosis in patients with severe virus-induced diseases. Some T cell subsets participate in tissue repair and wound healing (152, 153) and are useful for tissue engineering. For instance, the recruitment of antigen-specific T cells followed by Th2 adjuvant vaccination can help biomaterials for tissue repair (154). Studies on the influence of T cells on fibrosis are especially useful for the treatment of hypertrophic scarring, which is a severe form of fibrosis frequently developed in case of severe burn injuries. In early studies, T cells were found to be heavily infiltrated into the dermis and epidermis of human patients (155). In burn patients with hypertrophic scars, Th1 and Th2 cell subsets and cytokines were identified to be strongly associated with the development of fibrosis (156, 157). In a previous study, TGFβ-producing T cells were found in burn patients (158); Th22 cells were also found to promote fibroblast-mediated wound repair in an acute skin wounding mouse model (159). These T cells interact with and remodel ECM and shape the local immune and fibrogenic responses in both the epidermis and dermis of hypertrophic tissues (160). The initial trigger event for T cell activation and the crosstalk between T cells and keratinocytes, fibroblast, and epithelial cells thus become critical questions for resolving the niche and local environment. Although these types of studies are largely limited in human patients, several animal models have been developed to serve as powerful tools to study human hypertrophic scarring. Momtazi et al. grafted human skin onto several immune-deficient mice, including TCRαβ^−/−^γδ^−/−^, RAG-1^−/−^, and RAG-2^−/−^γc^−/−^ mice. The proliferative scars showed histological and immunohistochemical similarities to human hypertrophic scars. The study therefore not only proves the importance of T cells in scar formation but also provides a useful tool for human study (161). Similar studies were also performed with nude mice and SCID pig as animal models for studying the roles and functions of T cells in hypertrophic scar tissue (162, 163). Moreover, given the fact that T cells and T cell associated cytokines and chemokines differ over the period of scar formation (164), the findings of these animal model studies are particularly important for studying the temporal and spatial characteristic of fibrosis in terms of T cell-shaped local environment.

Are there novel pathways and mechanisms that are essential for T cell-mediated fibrotic diseases? How do we render T cells capable of halting the irreversible fibrotic response? There have been all kinds of approaches and clinical trials for fibrosis-related diseases (25, 165). T cell function can be modulated by lowering the level of cytokines, such as IFNγ (166), or by inhibiting kinases, such as hyperactivated focal adhesion kinase (FAK) (167). In some cases, altering the intestinal microbiota can reduce fibrosis (168). The checkpoint inhibitor for cancer therapy has drawn extensive attention in recent years and is also a promising way for fibrosis remodeling. The blockade of costimulatory signals such as OX40L or CTLA4 prevents fibrosis and induces the regression of established fibrosis (169, 170). Targeting a metabolic regulator is a prominent strategy for fibrosis reduction (171). Furthermore, mitochondrial biogenesis and endoplasmic reticulum (ER) stress have been associate with fibrosis (172, 173). Obesity-associated oxidative stress also contributes to fibrosis and fibrotic diseases (174). Accordingly, discoveries on fibrosis mechanisms warrant new opportunities to develop metabolic reprogramming drugs.

Technological advances shed light on the research as well as approaches for controlling fibrotic diseases. Single-cell sequencing provides the necessary tools to delineate the transcriptomic profiles of all types of individual cells. Thus, it has become possible to identify subpopulations that are critical for fibrogenesis and reveal new fibrogenic pathways (175). Chimeric antigen receptor (CAR) T cell therapy has been used in cancer treatment. Further, T cells engineered with fibroblast activation protein CAR have shown great potential to ablate cardiac fibroblasts, and significantly reduce cardiac fibrosis (176). In summary, this review discusses recent notable studies, and provides a framework for T cell-mediated fibrosis paving the way for rational targets and effective immunotherapies.

## Author Contributions

MZ prepared and wrote the manuscript. SZ edited the manuscript. All authors contributed to the article and approved the submitted version.

## Conflict of Interest

The authors declare that the research was conducted in the absence of any commercial or financial relationships that could be construed as a potential conflict of interest. The handling editor declared a past co-authorship with one of the authors SZ.
